# Specificity of the IgG antibody response to *Plasmodium falciparum*, *Plasmodium vivax*, *Plasmodium malariae*, and *Plasmodium ovale* MSP1_19_ subunit proteins in multiplexed serologic assays

**DOI:** 10.1186/s12936-018-2566-0

**Published:** 2018-11-09

**Authors:** Jeffrey W. Priest, Mateusz M. Plucinski, Curtis S. Huber, Eric Rogier, Bunsoth Mao, Christopher J. Gregory, Baltazar Candrinho, James Colborn, John W. Barnwell

**Affiliations:** 1 Division of Foodborne, Waterborne, and Environmental Diseases at the Centers for Disease Control and Prevention, Atlanta, GA USA; 2 Division of Parasitic Diseases and Malaria, Centers for Disease Control and Prevention, Atlanta, GA USA; 3 US President’s Malaria Initiative, Centers for Disease Control and Prevention, Atlanta, GA USA; 4 University of Health Sciences, Phnom Penh, Cambodia; 5 Division of Vector-borne Diseases, Centers for Disease Control and Prevention, Fort Collins, CO USA; 6 National Malaria Control Programme, Maputo, Mozambique; 7 Clinton Health Access Initiative, Boston, MA USA

**Keywords:** Serology, Malaria, MSP1_19_, Multiplex, Specificity

## Abstract

**Background:**

Multiplex bead assays (MBA) that measure IgG antibodies to the carboxy-terminal 19-kDa sub-unit of the merozoite surface protein 1 (MSP1_19_) are currently used to determine malaria seroprevalence in human populations living in areas with both stable and unstable transmission. However, the species specificities of the IgG antibody responses to the malaria MSP1_19_ antigens have not been extensively characterized using MBA.

**Methods:**

Recombinant *Plasmodium falciparum* (3D7), *Plasmodium malariae* (China I), *Plasmodium ovale* (Nigeria I), and *Plasmodium vivax* (Belem) MSP1_19_ proteins were covalently coupled to beads for MBA. Threshold cut-off values for the assays were estimated using sera from US citizens with no history of foreign travel and by receiver operator characteristic curve analysis using diagnostic samples. Banked sera from experimentally infected chimpanzees, sera from humans from low transmission regions of Haiti and Cambodia (N = 12), and elutions from blood spots from humans selected from a high transmission region of Mozambique (N = 20) were used to develop an antigen competition MBA for antibody cross-reactivity studies. A sub-set of samples was further characterized using antibody capture/elution MBA, IgG subclass determination, and antibody avidity measurement.

**Results:**

Total IgG antibody responses in experimentally infected chimpanzees were species specific and could be completely suppressed by homologous competitor protein at a concentration of 10 μg/ml. Eleven of 12 samples from the low transmission regions and 12 of 20 samples from the high transmission area had antibody responses that were completely species specific. For 7 additional samples, the *P. falciparum* MSP1_19_ responses were species specific, but various levels of incomplete heterologous competition were observed for the non-*P. falciparum* assays. A pan-malaria MSP1_19_ cross-reactive antibody response was observed in elutions of blood spots from two 20–30 years old Mozambique donors. The antibody response from one of these two donors had low avidity and skewed almost entirely to the IgG_3_ subclass.

**Conclusions:**

Even when *P. falciparum*, *P. malariae*, *P. ovale*, and *P. vivax* are co-endemic in a high transmission setting, most antibody responses to MSP1_19_ antigens are species-specific and are likely indicative of previous infection history. True pan-malaria cross-reactive responses were found to occur rarely.

**Electronic supplementary material:**

The online version of this article (10.1186/s12936-018-2566-0) contains supplementary material, which is available to authorized users.

## Background

Approximately 2.5 billion people, or one-third of the world’s estimated 2018 population live in regions of stable or unstable malaria transmission, and are at risk for infection [1, 2]. In sub-Saharan Africa, *Plasmodium falciparum* has been the major focus of treatment and intervention strategies because of the high mortality associated with infection. Three additional species of human malaria, two *Plasmodium ovale* sub-species and *Plasmodium malariae*, share much of the same geographic range in Africa yet are considered less important because prevalence estimates based on microscopic detection of parasites in blood films are generally low [3]. However, mounting clinical evidence suggests that malaria infection with species other than *P. falciparum* is not benign and that infection prevalence may be increasing in children, even in areas where anti-malarial drug therapy is regularly administered [4–8]. Similarly, the risk of *Plasmodium vivax* infection in sections of sub-Saharan and central Africa has been considered to be nil because large fractions of the populations in these regions lack the Duffy receptor used by the parasite for red blood cell invasion [9–12]. New evidence, however, suggests that low levels of *P. vivax* transmission in Africa may be occurring in susceptible Duffy-positive residents and that some level of infection is also occurring in Duffy-negative individuals by another reticulocyte invasion mechanism [13–16]. Because these non-*P. falciparum* infections are frequently sub-patent and their symptoms may be masked by the overwhelming levels of *P. falciparum* parasitaemia, accurate mapping and the estimation of prevalence levels in this population are difficult using traditional microscopic or PCR methods.

Serologic assays that detect IgG antibodies to specific *P. falciparum* and *P. vivax* antigens have been used in multiple studies in many parts of the world to estimate infection incidence and immunity levels (reviewed in [17–19]). Antibody data from cross-sectional surveys can be used to calculate the community-level seroconversion rates [20–27], and longitudinal and cross-sectional data provide similar estimates of community seroconversion rates [28]. Serologic assays using species-specific antigens could identify individuals who either are currently infected or have been previously infected with different malaria species, even if the infections were sub-patent [29]. Advances in multiplex assay technology make serologic antibody assays for multiple malaria antigens more attractive because antibody responses to a range of malaria antigens can be detected in a single well from a small volume of blood or serum and because malaria-specific assays can be integrated with assays for other antibody responses of public health interest [30–33].

One target antigen frequently used in malaria serologic antibody studies is the 19-kDa carboxy-terminal sub-unit of the merozoite surface protein 1 (MSP1_19_) [34–36], a glycosylphosphatidylinositol-anchored fragment of the larger MSP1 protein that is found in abundance on the parasite surface (reviewed in [37]). Although the MSP1_19_ proteins from *P. falciparum*, *P. malariae*, *P. ovale*, and *P. vivax* share 48–58% identity at the amino acid level [38], many of the conserved residues are cysteines and other hydrophobic amino acids that are unlikely to be exposed to the immune system [39]. Despite the sequence similarity, Cook et al. [24] were able to demonstrate unique seroprevalence curves for the *P. falciparum* and *P. vivax* MSP1_19_ antibody responses in areas of reduced transmission in Vanuatu. Similarly, Bousema et al. [40] did not observe any correlation between the *P. falciparum* and *P. vivax* MSP1_19_ antibody responses in ELISA studies of sera from a population living in a Somalian region of low endemicity for both parasites. In a study of malaria antibody responses in adult Cambodian women, Priest et al. [33] found that 79% of sera from women who were positive for antibodies to malaria reacted with the MSP1_19_ antigen from only one species. However, all of these three studies were conducted in regions of relatively low transmission, and it is important to determine whether the MSP1_19_ antigen-based assays will be species specific in regions of high transmission with multiple circulating species of malaria parasite.

During a bed net intervention study in a high malaria transmission region of northern Mozambique [41], numerous samples from individuals were assayed and found to have very high IgG antibody responses to multiple malaria species MSP1_19_ antigens, including the *P. vivax* antigen. These samples and samples from two low transmission regions in a multiplex assay format to expand on the MSP1_19_ competition ELISA studies of Amanfo et al. [42].

## Methods

### Human sample sets

Anonymous serum samples (N = 88), collected prior to 2000 from US citizens with no history of foreign travel, were presumed to be negative for antibodies to *Plasmodium* spp. and were used to define the cut-off values for the various assays. Anonymized, residual sera submitted to the Centers for Disease Control and Prevention between 1995 and 2011 for malaria diagnostic testing were used for the assessment of multiplex assay sensitivity. The panel included sera from patients having microscopically confirmed and/or PCR confirmed infections with *P. falciparum* (N = 33), *P. malariae* (N = 6), *P. ovale* (N = 7), or *P. vivax* (N = 35) [43]. The timing of sample collection relative to malaria infection or symptom development was not known. In addition to a pan-*Plasmodium* spp. immunofluorescence assay (IFA) positive serum pool (CDC Lot 8), mono-specific infection IFA serum controls were available for *P. falciparum* (CDC Lot 6) and *P. malariae* (CDC Lot 2).

Sera or dried blood spots previously identified by multiplex assay as having high levels of IgG antibodies to one or more MSP1_19_ proteins were selected for the specificity studies. This set included: 3 anonymous, adult blood donor samples collected in 1998 from a region of Haiti with a low prevalence of *P. falciparum* infection [43]; 9 sera from an integrated serologic study of immune status to vaccine-preventable diseases and neglected tropical diseases conducted in 2012 among women 15–39 years of age in Cambodia [33, 44, 45]; and, 20 dried blood spots from participants (4–60 years of age) in a long-lasting insecticide-treated bed net impact study conducted in 2013–2014 in a high malaria transmission province of northern Mozambique [41]. The sample set from Mozambique was biased towards individuals with a positive antibody response to the *P. vivax, P. ovale* and *P. malariae* antigens.

### Ethics statement

Residual malaria diagnostic sera were made anonymous under a protocol approved by the CDC Institutional Review Board. Written informed consent was obtained prior to enrolment and participation in the Cambodian sero-survey, and the study protocol was reviewed and approved by the National Ethics Committee in Cambodia [33, 44, 45]. Written informed consent was obtained prior to enrolment and participation in the Mozambique bed net study and sero-survey, and the study was approved by the National Bioethics Committee in Mozambique. For both of these studies, CDC researchers had no access to personal identifiers, and CDC staff were not considered to be engaged with human research subjects.

### Banked chimpanzee sera

Banked sera from malaria studies conducted in experimentally infected chimpanzees prior to 2000 were included in this report. Chimpanzees Bit and Klimatis were infected with the Uganda I strain of *P. malariae* [46, 47], Alpert was infected with the Nigeria I strain of *P. ovale* [48], and Duff was infected with the Salvador I strain of *P. vivax* [49]. As previously described, all animals had been splenectomized before they were inoculated intravenously with heparinized, infected blood.

### Antigens for multiplex assay

The cloning of the 3D7 strain *P. falciparum* MSP1_19_ in pGEX 4T-2 plasmid (GE Healthcare, Piscataway, NJ, USA) as a fusion protein with *Schistosoma japonicum* glutathione-*S*-transferase (GST) and the purification of the MSP1_19_-GST fusion protein have been described elsewhere [50].

Using the protocol of Priest et al. [33] and a new reverse PCR primer, a *P. vivax* MSP1_19_ clone lacking the carboxy-terminal, hydrophobic anchor sequence was generated in pGEX 4T-2 plasmid (GE Healthcare), and the MSP1_19_-GST fusion protein was purified. The target sequence was amplified from Belem strain DNA using a reverse deoxyoligonucleotide PCR primer with the following sequence: 5′-GCG GAA TTC *TTA* GCT GGA GGA GCT ACA GAA AAC TCC C-3′. The underlined sequence reverse primer identifies an *Eco*RI restriction endonuclease site used in directional cloning, and the italicized bases identify an introduced in-frame stop codon. All other cloning conditions remained as previously described [33]. The clone was sequenced using BigDye Terminator V3.1 chemistry (Applied Biosystems/Thermo Fisher Scientific, Foster City, CA, USA).

Cloning, expression and purification of a *P. ovale* MSP1_19_-GST fusion protein from Nigeria I strain DNA was accomplished using the strategy described in Priest et al. [33] with the following deoxyoligonucleotide primers: forward, 5′-CGC GGA TCC TCT ATG GGA TCT AAA CAT AAA TGT-3′ and reverse, 5′-GCG GAA TTC *TTA* ACT TGA TGA GCC ACA GAA AAC ACC-3′. The underlined sequence in the forward primer identifies a *Bam*HI restriction endonuclease site used in directional cloning. These primer sequences were based on the sequence of the Cameroon OMA1A *P. ovale* isolate sequence (GenBank accession number FJ824670) described by Birkenmeyer et al. [38].

Cloning of the *P. malariae* MSP1_19_ coding sequence from China I strain DNA required two PCR reactions. The first reaction used long PCR primers (forward, 5′-AAT ATT AGC GCA AAA CAT GCA TGT ACC GAA ACA-3′; reverse, 5′-ACT TGA AGA ACC ACA GAA AAC ACC TTC AAA TAT AG-3′) and the amplification conditions previously described [33]. These primer sequences were based on the sequence of the Cameroon MM1A *P. malariae* isolate sequence (GenBank accession number FJ824669) described by Birkenmeyer et al. [38]. A total of 5% of the purified primary product (StrataPrep PCR purification kit, Stratagene, LaJolla, CA, USA) was used in a second amplification reaction with the following primers: forward, 5′-CGC GGA TTC AAT ATT AGC GCA AAA CAT GCA TGT-3′; reverse, 5′-GCG GAA TTC *TTA* ACT TGA AGA ACC ACA GAA AAC ACC-3′. This final PCR product was cloned in pGEX 4T-2 plasmid (GE Healthcare), and a GST fusion protein was expressed and purified using the protocol of Priest et al. [33].

Expression and purification of the control GST protein with no fusion partner has been described elsewhere [51]. A synthetic 20 amino acid peptide, (NANP)_5_-amide, corresponding to the carboxy-terminal repeat of the *P. falciparum* circumsporozoite protein (PfCSP peptide) [52, 53] was cross-linked to GST using the glutaraldehyde protocol of Benitez et al. [54]. Tetanus toxoid antigen from Massachusetts Biologic Laboratories (Boston, MA, USA) was exchanged into buffer containing 10 mM Na_2_HPO_4_ and 0.85% NaCl at pH 7.2 (PBS) [44].

### Comparison of *Plasmodium malariae* MSP1_19_ sequences from other geographic locations

Ten nanograms of DNA from *P. malariae* strains Greece I, Guyana, and Uganda I were PCR amplified using the forward and reverse long deoxyoligonucleotides described above and the Expand High Fidelity PCR system (Roche Applied Science, Indianapolis, IN, USA). Cycle conditions were as follows: 94 °C for 5 min, 35 cycles of 95 °C for 30 s, 55 °C for 30 s, and 68 °C for 1 min, and a final extension step of 68 °C for 5 min. Products were purified (StrataPrep PCR purification kit, Stratagene) and sequenced as described above.

### Antigen coupling and multiplex bead assays

Antigens were coupled in 1.0 ml of buffer containing 25 mM 2-(*N*-morpholino)-ethanesulfonic acid (MES) at pH 5.0 with 0.85% NaCl using the following amounts of protein for 12.5 × 10^6^ SeroMap microspheres (Luminex Corp, Austin, TX, USA): MSP1_19_-GST fusion proteins, 30 μg; GST control protein, 15 μg; PfCSP peptide-GST, 30 μg; and tetanus toxoid, 12.5 μg. The coupling protocol and bead storage buffer have been described previously [55].

Blood spots were collected on filter paper disks (Cellabs, Sydney, Australia). A single tab containing 10 μl whole blood (approximately 5 μl of serum) was eluted overnight at 4 °C in 200 μl of buffer containing PBS with 0.05% Tween-20 and 0.05% NaN_3_ for a 1:40 serum protein dilution [56]. Samples were further diluted 1:10 (final 1:400 serum dilution) in PBS buffer (pH 7.2) containing 0.3% Tween-20, 0.02% NaN_3_, 0.5% casein, 0.5% polyvinyl alcohol (PVA), 0.8% polyvinylpyrrolidone (PVP), and 3 μg/ml *Escherichia coli* extract (Buffer A) [33, 57]. Test sera were diluted 1:400 in Buffer A. BSA was not included in the dilution buffer as it was found to be unnecessary for blocking when casein was present.

The multiplex bead assay protocol for total IgG has been described elsewhere [55, 58]. Assays were run in duplicate wells, and each plate included a buffer only blank. The reported “median fluorescent intensity *minus* background” value (MFI-bg) is the average of the 2 median fluorescent intensity values *minus* background blank values from two replicate wells. Negative MFI-bg values were set to 0.

In the multiplex IgG sub-class assays, serum antibodies were bound to beads using the previously described multiplex assay protocol [55, 58]. Washed beads were then incubated for 45 min at room temperature with 50 μl/well of a 1:500 dilution in Buffer B (0.5% BSA, 0.05% Tween-20, and 0.02% NaN_3_ in PBS at pH 7.2) of biotinylated monoclonal mouse anti-human IgG subclass secondary antibody to IgG_1_ (clone HP6025), IgG_2_ (clone HP6002), IgG_3_ (clone HP6047), or IgG_4_ (clone HP6025) (all from Zymed/Invitrogen, South San Francisco, CA, USA). Wells were developed with *R*-phycoerythrin-labelled streptavidin and read on a BioPlex 200 instrument (BioRad, Hercules, CA, USA) as described above.

### Assessment of coupling efficiency

To determine whether the *Plasmodium* spp. GST-MSP1_19_ fusion proteins were coupled to the SeroMap beads with similar efficiencies, multiplex assays were run using a serial dilution of a goat anti-GST polyclonal IgG antibody (GE Healthcare) as the primary antibody to detect the fusion protein on the bead. The initial dilution of anti-GST antibody was 1:1000 in modified Buffer A lacking the *E. coli* extract (50 μl/well), and the final dilution was 1:1.0 × 10^7^. Bound anti-GST antibody was detected with 50 μl/well of a biotinylated rabbit anti-goat IgG secondary antibody (1:500 dilution in Buffer B; Invitrogen) and wells were developed with *R*-phycoerythrin-labelled streptavidin and read on a BioPlex 200 instrument (BioRad) as described above.

### MSP1_19_ competition assays

Serial dilutions of purified MSP1_19_-GST competitor proteins were generated from a 0.5 mg/ml stock solution using PBS buffer at pH 7.2. A 96-well incubation plate (V-bottom, Fisher Scientific) was set up such that wells contained 3 μl of the competitor MSP1_19_-GST fusion protein dilution and 147 μl of serum dilution in Buffer A for 1:50 dilution of competitor protein and a negligible further dilution of the serum. Final competitor protein concentrations in the serum dilution ranged from 10 μg/ml to as low as 0.025 μg/ml. The plate was incubated at room temperature for 1 h, and then each well of the incubation plate was used to load duplicate multiplex bead assay wells at 50 μl each. The standard total IgG assay protocol was then followed as described above. The standard MSP1_19_ competition assay used a final competitor protein concentration in diluted serum of 10 μg/ml, and a ≥ 30% reduction in multiplex assay signal was considered to be evidence of antibody cross-reactivity.

### MSP1_19_-specific antibody binding and elution

Using the standard coupling protocol [55], individual MSP1_19_-GST fusion proteins were coupled to magnetic beads (region 14, Luminex) in 100 μl of MES/NaCl buffer at pH 5.0 at 4.5 μg for 1.25 × 10^6^ microspheres (a 50% increase in protein compared to SeroMap bead amounts). Coupled beads were re-suspended in 120 μl of storage buffer with protease inhibitors [55]. A 1:200 dilution of serum in Buffer A or a 1:5 dilution of blood spot eluate in Buffer A (approximately 1:200 serum dilution) was incubated for 1 h at room temperature with 20 μl of coupled beads (washed 1× with 0.05% Tween-20 in PBS prior to use). Beads were collected by magnetic capture, the used serum or blood spot dilution was removed, and the beads were washed 4× with 200 μl 0.05% Tween-20 in PBS. To elute the bound antibodies, beads were re-suspended for 10 min at room temperature in 100 μl of buffer containing 3 parts of 4 M MgCl_2_ in 100 mM *N*-hydroxyethylpiperazine-*N*′-2-ethanesulfonic acid (HEPES) at pH 8.0 and 1 part ethylene glycol [59]. The beads were collected by magnetic capture, and the supernatant was removed and diluted into 0.9 ml of buffer containing 50 mM tris(hydroxymethyl)-aminomethane (Tris) at pH 7.5 and 0.85% NaCl. The antibody elution process was repeated once. The 2 ml of eluted antibody in Tris/NaCl buffer was concentrated to 50 μl using a Centricon-50 centrifugal filter device as directed by the manufacturer (Millipore, Bedford, MA, USA). The concentration procedure was repeated following a 2 ml Tris/NaCl buffer dilution and again after a 1 ml PBS buffer dilution. The final 30–50 μl of concentrate was diluted with > 3 volumes of Buffer A, and duplicate multiplex assays were performed using half of the eluted antibody per well.

### Antibody avidity determinations

To determine the avidity of IgG antibody binding, MSP1_19_-GST fusion protein coated SeroMap beads incubated for 1 h with 1:400 serum dilutions were immediately washed with 100 μl of 6 M urea in PBS for 5 min at room temperature [60]. The urea wash was repeated once followed by three 100 μl washes with 0.05% Tween-20 in PBS. The normal total IgG development protocol was then followed. An avidity index was calculated by dividing the 6 M urea-treated MFI-bg value by the untreated MFI-bg value.

### Data analysis

Protein sequences were aligned using COBALT [61]. The means plus 3 standard deviations of the MBA responses from 88 adult US citizens with no history of foreign travel were used to define potential cutoffs for the MSP1_19_ protein and CSP peptide assays. Receiver-operating characteristic (ROC) curves were also used to generate potential cut-offs for the MSP1_19_ assays. The ROC analysis [62] and Spearman rank order correlation analysis were performed using SigmaPlot 13.0 (Systat Software, Inc., San Jose, CA, USA). The *J*-index [63] was calculated from the sensitivity and specificity values.

## Results

### MSP1_19_ sequences

**Fig. 1:**
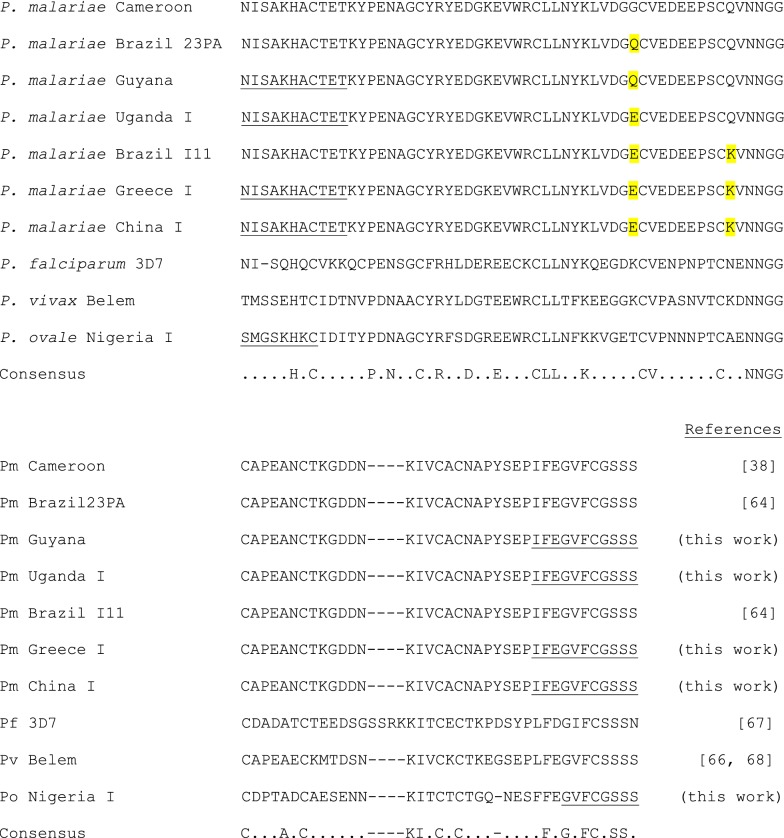
Alignment of predicted *Plasmodium* spp. MSP1_19_ protein sequences using COBALT [61]. Residues in the *P. malariae* sequence that differ from the Cameroon sequence of Birkenmeyer et al. [38] are shaded. Predicted protein sequences resulting from the oligonucleotides used in PCR amplification are underlined. The positions of residues conserved among all the presented MSP1_19_ protein sequences are indicated in the consensus with divergent residues indicated by a dot. GenBank accession numbers are MH577181, *P. ovale* Nigeria I strain; MH577182, *P. malariae* China I strain; MH577183, *P. malariae* Greece I strain; MH577184, *P. malariae* Uganda I strain; and MH577185, *P. malariae* Guyana strain

The DNA sequence of the *P. malariae* China I strain MSP1_19_ clone (deposited in GenBank as MH577182) differed from the Cameroon sequence of Birkenmeyer et al. [38] at 3 nucleotide base positions, leading to 2 amino acid substitutions in the deduced amino acid sequence: G41E and Q51K (numbering based on mature MSP1_19_ protein sequence). As shown in Fig. 1, the deduced amino acid sequence of the China I strain was identical to that reported for the Brazil I11 strain [64] and was also identical to that of the Greece I strain (GenBank MH577183). Compared to the Cameroon strain, the Uganda I strain of *P. malariae* contained only a G41Q amino acid substitution (GenBank MH577184), while the Guyana strain contained only a G41E substitution (GenBank MH577185).

The DNA sequence of the *P. ovale* Nigeria I clone (GenBank MH577181) matched the GenBank sequence reported for the Cameroon OMA1A *P. ovale* isolate (FJ824670) by Birkenmeyer et al. [38]. The Nigeria I strain likely belongs to the newly identified *Plasmodium ovale curtsi* species as the MSP1_19_ predicted protein sequence has a Ser at position 23 rather than a Pro [65]. The sequence of the *P. vivax* clone matched that found in GenBank (accession number AF435594.1) [66]. Alignment of the deduced MSP1_19_ amino acid sequences of the *P. malariae*[38, 64], *P. falciparum* 3D7 strain [67], *P. vivax* Belem strain[66, 68], and *P. ovale* Nigeria I strain proteins in Fig. 1 showed conservation of 32 amino acids among the four species including 10 cysteines and 5 hydrophobic residues.

### Assessment of coupling efficiency

**Fig. 2:**
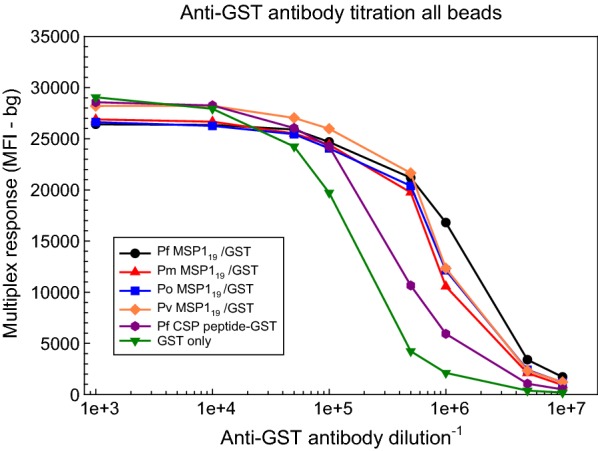
Assessment of coupling efficiency using a dilution series of goat anti-GST IgG antibody. Goat anti-GST IgG antibody was diluted 1:1 × 10^3^, 1:1 × 10^4^, 1:5 × 10^4^, 1:1 × 10^5^, 1:5 × 10^5^, 1:1 × 10^6^, 1:5 × 10^6^, and 1:1 × 10^7^ in modified Buffer A lacking the *E. coli* extract. Following a 1-h incubation (50 μl/well) at room temperature, bound anti-GST antibody was detected with a biotinylated rabbit anti-goat IgG secondary antibody (1:500 dilution in Buffer B, 50 μl/well, 1 h at room temperature). Wells were developed with *R*-phycoerythrin-labelled streptavidin and read on a BioPlex 200 instrument (BioRad) as described in “Methods”

The multiplex response titration curves using dilutions of the anti-GST antibody as the primary antibody in the multiplex reaction were similar for all 4 proteins (Fig. 2). In contrast, the multiplex response titration curves for the GST control bead (coupled at half the protein concentration of the MSP1_19_-GST reactions) and for the cross-linked *P. falciparum* CSP peptide-GST bead were indicative of lower amounts of bound target protein compared to the MSP1_19_ beads.

### Cut-off determinations

One outlier from the group of 88 US citizens with no history of foreign travel with a MBA response of 8690 MFI-bg units was censored from the *P. vivax* cut-off calculation, and one outlier with a MBA response of 10,377 MFI-bg units was censored from the *P. falciparum* CSP peptide calculation. The cut-offs in MFI-bg units were: *P. falciparum* CSP peptide, 1351; *P. falciparum* MSP1_19_, 313; *P. malariae* MSP1_19_, 397; *P. ovale* MSP1_19_, 65; and, *P. vivax* MSP1_19_, 86. In an analysis of the residual diagnostic serum panel that included sera from patients having microscopically confirmed and/or PCR confirmed infections, 26 of 33 (79%) of *P. falciparum*, 5 of 6 (83%) of *P. malariae*, 5 of 7 (71%) *P. ovale*, and 33 of 35 (94%) of *P. vivax* were positive by multiplex assay. The sensitivity of the *P. falciparum* CSP peptide assay was not determined. Specificities measured from the presumed negative US citizen panel were ≥ 96% for each assay.

Cut-offs determined from all MSP1_19_ values (no outliers censored) using ROC curve analysis [62] were lower for *P. falciparum* and *P. malariae* (111 and 237 MFI-bg units, respectively) but higher for *P. ovale* and *P. vivax* (175 and 203 MFI-bg units, respectively). *J*-index analysis [63] yielded identical cut-off values. The ROC cut-off values had no impact on the sensitivities of the assays for *P. malariae*, *P. ovale* or *P. vivax* and either had no impact (*P. malariae*) or resulted in increases of 3% (*P. ovale*) or 2% (*P. vivax*) in specificity. Sensitivity and specificity for the *P. falciparum* assay using the ROC cut-offs were 88 and 94%, respectively.

In order to maximize specificity and to estimate seropositivity conservatively, the higher of the cut-off values from the various methods of analysis for the MSP1_19_ assays (in MFI-bg units) were chosen: *P. falciparum* MSP1_19_, 313; *P. malariae* MSP1_19_, 397; *P. ovale* MSP1_19_, 175; and, *P. vivax* MSP1_19_, 203.

### MSP1_19_ multiplex assay specificity

If closely related antigens coupled on different beads share common epitopes and compete for the same pool of antibodies, the values from a multiplexed assay would be expected to be lower than the values from assays using a single bead only. To test this hypothesis, each *Plasmodium* spp. MSP1_19_ was assayed in isolation (individual monoplex), and the results were compared to values obtained when all of the beads were included in the routine multiplex format. As shown in Table 1, responses from 2 defined sera (Pan *Plasmodium* spp. Lot 8 and *P. malariae* Lot 2), 3 elutions from individual Mozambique blood spots, and one high-titre elution from a combination of Mozambique blood spots were essentially identical regardless of the bead complexity of the assay (Spearman rank order correlation coefficient = 0.999; *P* < 0.001). The results from this limited panel of samples suggest that a response dilution effect in the multiplex assay format is not a universal feature of the MSP1_19_ protein family and that the multiplex assay may be useful in infection species determinations.

**Table 1:** Impact of bead complexity on multiplex bead assay response values using beads coated with MSP1_19_ proteins from four malaria species

Sample	Assay type	Pf^1^ MSP1_19_ (MFI-bg)	Pm MSP1_19_ (MFI-bg)	Po MSP1_19_ (MFI-bg)	Pv MSP1_19_ (MFI-bg)	Pf CSP-peptide (MFI-bg)	GST (MFI-bg)
Pan *Plasmodium* Lot 8 serum	Individual monoplex	28,537	355	721	23,449	N/A	N/A
	Multiplex	28,442	351	724	23,688	2281	41
*P. malariae* Lot 2 serum	Individual monoplex	19	10,413	6	2	N/A	N/A
	Multiplex	20	10,878	6	2	170	11
Mozambique donor 15	Individual monoplex	28,088	1437	16,504	260	N/A	N/A
	Multiplex	28,242	1446	17,352	272	26,039	53
Mozambique donor 7	Individual monoplex	9396	734	9566	78	N/A	N/A
	Multiplex	9484	798	10,623	86	22,359	11
Mozambique donor 8	Individual monoplex	28,034	23,220	11	45	N/A	N/A
	Multiplex	28,438	23,378	14	47	15,819	11
Mozambique elution mix	Individual monoplex	28,842	27,229	20,193	24,113	N/A	N/A
	Multiplex	28,911	27,082	21,585	24,048	17,682	279

*N/A* assay not performed

That some MSP1_19_ antibody responses are species specific can also be demonstrated using sera from experimentally infected chimpanzees (Table 2). Sera from chimpanzees Klimatis (*P. malariae* infection) and Duff (*P. vivax* infection) had high antibody response values to the corresponding species-specific MSP1_19_ protein and had no responses to MSP1_19_ antigens from other species. In contrast, other animals such as chimpanzees Alpert (*P. ovale* infection) and Bit (*P. malariae* infection) reacted strongly with the MSP1_19_ antigen corresponding to the species of the infecting parasite, but they also had weak responses to *P. vivax* MSP1_19_ and strong responses to *P. falciparum* MSP1_19_. The wild-caught chimpanzees used in the experimental infection studies were never exposed to *P. falciparum* sporozoites in the laboratory and were never experimentally infected with *P. falciparum*. Thus, the presence of a *P. falciparum* CSP peptide response suggests that the *P. falciparum* MSP1_19_ response likely arose by natural infection with a closely related species of chimpanzee malaria, such as *Plasmodium reichenowi* [69], rather than with an experimentally-induced cross-reactivity. Similarly, the weak *P. vivax* antibody responses in these 2 chimpanzees may also reflect prior exposure to *P. vivax*-like parasites in the wild. These unexpected responses highlight the difficulty of differentiating historic infection from true antibody cross-reaction.

**Table 2:** Multiplex bead assays results using sera from chimpanzees experimentally infected with a single species of malaria parasite

Chimpanzee name	Lab. infection species	Pm MSP1_19_ (MFI-bg)	Po MSP1_19_ (MFI-bg)	Pv MSP1_19_ (MFI-bg)	Pf MSP1_19_ (MFI-bg)	Pf CSP-peptide (MFI-bg)	GST (MFI-bg)
Klimatis	*P. malariae*	23,289	1	2	119	14	0
Duff	*P. vivax*	41	51	26,588	64	156	3
Alpert	*P. ovale*	71	22,581	724	25,757	4002	0
Bit	*P. malariae*	24,223	49	664	5247	23,270	19

### Specificity analysis using antigen competition

**Fig. 3:**
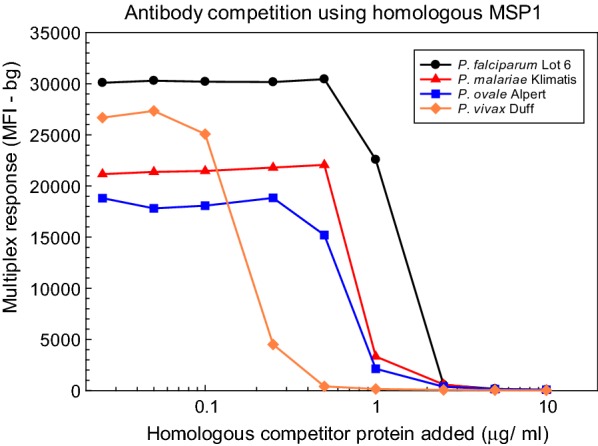
Antibody competition titration assays using homologous MSP1_19_ proteins. Dilutions (1:400) of *P. falciparum* Lot 6 defined human serum or of sera from chimpanzees experimentally infected with either *P. malariae* (Klimatis), *P. ovale* (Alpert) or *P. vivax* (Duff) were incubated with the indicated concentrations of the homologous MSP1_19_ competitor protein for 1 h at room temperature. Multiplex bead assays were performed as described in “Methods”, and the multiplex responses in MFI-bg units are plotted *versus* the competitor concentration

An alternative approach to assess the specificity of the MSP1_19_ antibody response relies on the ability of soluble antigen to saturate the antibody in a pre-incubation step so as to prevent antibody binding to MSP1_19_ coated beads during the multiplex assay. To determine the concentration of competitor protein necessary to prevent antibody binding to MSP1_19_ coated beads, sera that had high antibody responses were incubated with 0.025–10 μg/ml of competitor protein prior to multiplex assay as described in “Methods”. The GST control, PfCSP peptide-GST, and all 4 MSP1_19_-GST protein-coated beads were included in the multiplex assay, but only the homologous MSP1_19_ was used as competitor. Thus, the *P. falciparum* Lot 6 defined human serum was competed using soluble *P. falciparum* MSP1_19_-GST fusion protein, while sera from chimpanzees Klimatis (*P. malariae*), Alpert (*P. ovale*), and Duff (*P. vivax*) were each competed using the corresponding species-specific MSP1_19_-GST fusion proteins. Figure 3 shows that the multiplex responses to all 4 MSP1_19_ proteins were reduced > 97% following pre-incubation with 2.5 μg/ml competitor protein, and all 4 MSP1_19_ antibody responses were below their respective cut-off values when sera were pre-incubated with 5 μg/ml competitor protein.

**Fig. 4:**
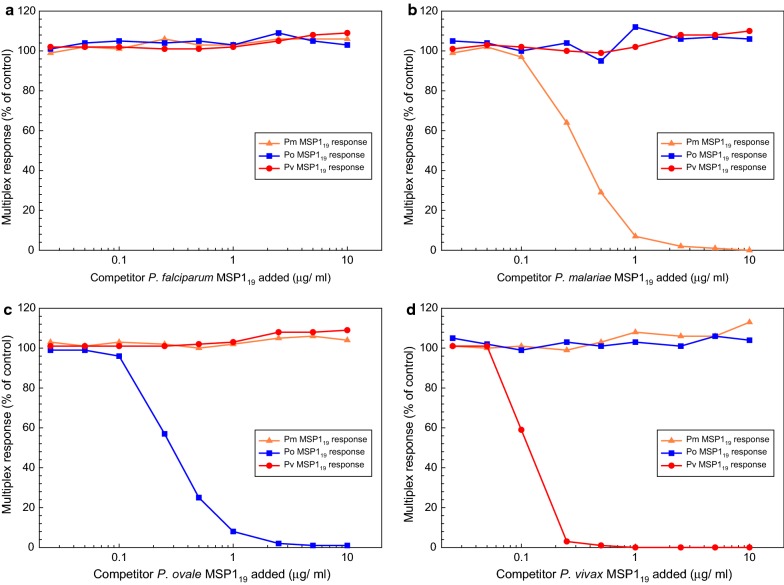
Antibody competition titration assays using MSP1_19_ proteins from four *Plasmodium* species. A combined dilution (1:400 of each serum) containing sera from chimpanzees experimentally infected with either *P. malariae* (Klimatis), *P. ovale* (Alpert) or *P. vivax* (Duff) was incubated with the indicated concentrations of the MSP1_19_ competitor protein for 1 h at room temperature. Competitor proteins used were: **a** *P. falciparum* MSP1_19_; **b** *P. malariae* MSP1_19_; **c** *P. ovale* MSP1_19_; **d** *P. vivax* MSP1_19_. Multiplex bead assays were performed as described in “Methods” and the multiplex response in MFI-bg units are plotted *versus* the competitor concentration. Multiplex responses are presented as a percentage of the assay results for the PBS control

Next, sera from chimpanzees Klimatis (*P. malariae*), Alpert (*P. ovale*), and Duff (*P. vivax*) (1:400 dilution of each serum) were combined, and the competitor titration assays were repeated. Figure 4 shows the multiplex responses in the presence of various concentrations of the 4 MSP1_19_ competitor proteins and expressed as a percentage of the PBS control. In Panel A, addition of the *P. falciparum* MSP1_19_ competitor protein had no effect on the *P. malariae*, *P. ovale* or *P. vivax* multiplex responses. Similarly, heterologous MSP1_19_ competitor proteins had no effect on multiplex responses (Fig. 4b–d), while multiplex response curves for the homologous species of competitor MSP1_19_ proteins in Fig. 4b–d resemble the individual curves previously shown in Fig. 3. The chimpanzee multiplex responses in the presence of homologous species competitor protein showed > 97% suppression at 2.5 μg/ml and were below the respective cut-off values at 5 μg/ml of competitor.

Finally, combined human sera (pan-*Plasmodium* spp. positive serum pool and *P. malariae* mono-specific infection serum control, each at 1:400 dilution) competitor studies showed similar heterologous and homologous titration profiles except that the *P. malariae* response was reduced by approximately 27–29% at the 10 μg/ml of heterologous MSP1_19_-GST competitor protein concentrations (Additional file 1). For the human multiplex responses in the presence of homologous species competitor protein, values were below the respective cut-off values at 2.5 μg/ml of competitor. Based on these studies, a competitor concentration of 10 μg/ml was selected to maximize suppression of antibody binding in the multiplex assay.

### Assay specificity in low malaria incidence settings

Representative competition assay results for a serum sample set from two regions of low malaria incidence (Haiti and Cambodia) are presented in Table 3. Additional results from this sample set can be found in Additional file 2. Most of the samples chosen from these areas had positive antibody responses to only one or two MSP1_19_ proteins, and only one person (Cambodia 5) reacted with MSP1_19_ proteins from three malaria species. Antibody responses to the *P. falciparum* CSP peptide were mostly negative, and, when present, were < 4000 MFI-bg units (median = 64.5; range 14–3930). Addition of GST control protein to the competition assay at a concentration of 10 μg/ml had no effect on any of the antibody responses. One person had an antibody response to the GST coupled control bead, but the response was not inhibited by pre-incubation with soluble GST protein. This response was probably unrelated to the presence of the GST protein as the *P. ovale* MSP1_19_-GST response was consistently < 50 MFI-bg units.

**Table 3:** Representative MSP1_19_ competition assay results using sera from low incidence settings

Sample	Competitor added	Pf MSP1_19_ (MFI-bg)	Pm MSP1_19_ (MFI-bg)	Po MSP1_19_ (MFI-bg)	Pv MSP1_19_ (MFI-bg)	Pf CSP (MFI-bg)	GST (MFI-bg)
Haiti 1	PBS buffer only	25,260	15	21	9	65	1
	GST	25,473	16	21	11	65	4
	Pf MSP1_19_	*13* ^a^	14	21	9	70	3
	Pm MSP1_19_	25,058	6	17	8	63	3
	Po MSP1_19_	25,344	12	6	7	62	2
	Pv MSP1_19_	25,181	11	18	4	65	3
Cambodia 2	PBS buffer only	35	21	15	23,836	64	1
	GST	37	22	17	23,623	66	1
	Pf MSP1_19_	28	24	11	21,057	70	1
	Pm MSP1_19_	40	13	17	23,348	61	2
	Po MSP1_19_	41	23	13	23,662	67	1
	Pv MSP1_19_	36	22	14	*9*	61	2
Cambodia 3	PBS buffer only	29,763	3177	10	9	2871	0
	GST	30,461	3443	12	14	2909	1
	Pf MSP1_19_	*32*	3428	11	15	2821	1
	Pm MSP1_19_	30,233	*9*	14	13	2817	0
	Po MSP1_19_	30,352	3480	10	12	3041	1
	Pv MSP1_19_	30,129	3043	12	4	2879	2
Cambodia 4	PBS buffer only	15	6	13	20,899	17	0
	GST	15	7	13	20,930	13	1
	Pf MSP1_19_	9	6	14	15,929	14	0
	Pm MSP1_19_	12	2	12	20,382	16	0
	Po MSP1_19_	11	5	2	20,177	15	0
	Pv MSP1_19_	13	6	12	*1*	13	0
Cambodia 9	PBS buffer only	1882	21	17	12,439	22	3
	GST	2023	23	21	13,095	21	4
	Pf MSP1_19_	*31*	22	23	9347	21	4
	Pm MSP1_19_	1877	13	23	12,075	20	4
	Po MSP1_19_	1854	24	17	12,679	21	2
	Pv MSP1_19_	1725	22	21	*4*	22	3
Cambodia 5	PBS buffer only	28,405	743	39	24,051	1072	800
	GST	28,084	799	44	24,307	1143	911
	Pf MSP1_19_	*50*	***439*** ^b^	34	23,510	1160	881
	Pm MSP1_19_	26,098	*31*	34	23,646	1136	839
	Po MSP1_19_	27,871	737	32	23,808	1103	861
	Pv MSP1_19_	27,620	617	33	*19*	1090	828

^a^Multiplex bead assay responses that were completely suppressed by homologous protein competition are indicated in italics cells. These responses were below the respective cut-off values

^b^Multiplex bead assay responses that, as a result of heterologous competition, decreased more than 30% compared to the PBS control but remained above the respective cut-off values are indicated in bold italics cells. These values indicate some level of cross-reactivity

In 9 of the 12 serum samples tested, all of the malaria MSP1_19_ antibody responses appeared to be species specific as only homologous competitor MSP1_19_ protein completely eliminated the antibody response (highlighted in italics in Table 3 and Additional file 2). For two of the tested sera (Table 3) multiplex assay response values to the *P. vivax* antigen demonstrated a weak heterologous competition effect with *P. falciparum* MSP1_19_ competitor protein, but the effect did not meet the 30% threshold definition (approximately 25% reduction; Cambodia 4 and 9). Interestingly, the sample from Cambodia donor 4 had no antibodies to either *P. falciparum* antigen by MBA. Only one donor had a heterologous competition assay response reduction of > 30%: for Cambodia 5 (indicated in bolditalics in Table 3), addition of *P. falciparum* MSP1_19_ competitor protein reduced a weak *P. malariae* response by 41% while completely eliminating a strong homologous *P. falciparum* MSP1_19_ response (> 28,000 MFI-bg units). The MFI-bg value for the heterologous competition assay remained above the respective *P. malariae* cut-off.

### Assay specificity in a high malaria incidence setting

Representative competition assay results for a sample set from a high malaria incidence region of Mozambique are presented in Table 4 with additional values shown in Additional file 3. These 20 samples were selected from the parent study [41] because they exhibited high IgG antibody responses to one or more MSP1_19_ antigens by multiplex bead assay, and the selection was biased towards samples that had a strong positive responses to the *P. vivax*, *P. ovale* or *P. malariae* proteins. Historically, rates of vivax malaria have been expected to be low in East African populations lacking the Duffy antigen [12], and an antibody response to the *P. vivax* MSP1_19_ might be indicative of antibody cross-reactivity. In contrast to the samples from low prevalence areas described above, eluted blood spot samples from Mozambique often had strong antibody responses to the *P. falciparum* CSP peptide (median = 23,407; range = 2833–27,033).

**Table 4:** Representative MSP1_19_ competition assay results using sera from a high incidence setting

Sample	Competitor added	Pf MSP1_19_ (MFI-bg)	Pm MSP1_19_ (MFI-bg)	Po MSP1_19_ (MFI-bg)	PvMSP1_19_ (MFI-bg)	PfCSP (MFI-bg)	GST (MFI-bg)
Mozambique 6	PBS buffer only	28,163	93	75	83	24,603	22
	GST	28,217	79	57	56	24,181	21
	Pf MSP1_19_	*45* ^a^	76	54	48	24,313	27
	Pm MSP1_19_	28,133	16	54	42	24,467	25
	Po MSP1_19_	28,237	71	32	47	24,533	21
	Pv MSP1_19_	27,881	71	57	17	24,367	25
Mozambique 13	PBS buffer only	29,635	24,263	660	28,869	26,738	6
	GST	29,480	24,168	656	28,481	26,414	6
	Pf MSP1_19_	*38*	24,276	594	28,799	26,792	6
	Pm MSP1_19_	29,729	*395*	618	29,039	26,728	6
	Po MSP1_19_	29,522	24,142	*29*	28,411	26,736	7
	Pv MSP1_19_	29,724	24,182	637	*122*	26,670	7
Mozambique 17	PBS buffer only	27,423	1082	23,326	87	23,363	2
	GST	27,420	1164	23,551	100	23,351	5
	Pf MSP1_19_	*25*	1189	23,599	104	23,481	3
	Pm MSP1_19_	27,280	*18*	23,389	97	23,514	2
	Po MSP1_19_	27,239	1157	*16*	94	23,468	4
	Pv MSP1_19_	27,199	1173	23,387	29	23,329	4
Mozambique 1	PBS buffer only	29,855	5009	4945	27,283	22,826	10
	GST	29,797	5191	4942	27,173	22,987	9
	Pf MSP1_19_	*66*	***3017*** ^b^	4998	26,901	22,818	11
	Pm MSP1_19_	29,636	*169*	4841	26,949	22,820	8
	Po MSP1_19_	29,597	4578	*44*	26,592	22,866	9
	Pv MSP1_19_	29,693	4946	4703	*57*	22,879	8
Mozambique 20	PBS buffer only	26,789	27,032	491	1227	23,870	24
	GST	26,841	26,891	536	1384	24,149	11
	Pf MSP1_19_	*98*	27,258	459	1301	24,083	30
	Pm MSP1_19_	26,668	488 ^c^	388	1084	23,921	20
	Po MSP1_19_	26,842	27,236	*29*	1033	24,089	26
	Pv MSP1_19_	26,785	27,183	352	*108*	23,745	22
Mozambique 16	PBS buffer only	27,518	6425	17,816	24,437	21,029	25
	GST	27,782	6588	18,263	24,430	21,019	17
	Pf MSP1_19_	*25*	***2040***	18,892	24,275	21,320	20
	Pm MSP1_19_	27,399	*80*	18,126	24,324	20,318	20
	Po MSP1_19_	27,866	6361	*113*	21,301	21,114	23
	Pv MSP1_19_	27,805	6029	***232***	2353	20,007	22
Mozambique 19	PBS buffer only	2677	2904	353	16,473	23,808	5
	GST	2977	3230	371	18,213	23,981	5
	Pf MSP1_19_	*12*	3187	366	17,993	24,120	4
	Pm MSP1_19_	2950	*40*	*151* ^d^	19,599	23,715	5
	Po MSP1_19_	2931	2605	*40*	***8192***	24,276	5
	Pv MSP1_19_	2648	2246	*107*	*50*	24,094	4

^a^Multiplex bead assay responses that were completely suppressed by homologous protein competition are indicated in italics cells. These responses were below the respective cut-off values

^b^Multiplex bead assay responses that, as a result of heterologous competition, decreased more than 30% relative to the PBS control but remained above the respective cut-off values are indicated in bold italics cells. These values indicate some level of cross-reactivity

^c^Multiplex bead assay responses that were only partially reduced by homologous protein competition are indicated in underlined cells. These responses remained above the respective cut-off values and likely represent incomplete antibody blocking

^d^Multiplex bead assay responses that were completely suppressed by heterologous protein competition are indicated in underlined italics cells. These responses were below the respective cut-off values

Species-specific anti-MSP1_19_ antibody responses, as indicated by the presence of complete homologous competition and the absence of heterologous competitor effects, were observed in 10 of the 20 samples tested (Table 4 and Additional file 3). In one additional sample (Mozambique 20 in Table 4), species-specific responses were observed, but the suppression of the *P. malariae* MSP1_19_-specific antibody responses was incomplete: values remained above the 397 MFI-bg assay cut-off threshold in the presence of 10 μg/ml *P. malariae* competitor protein. Given the very high *P. malariae* MSP1_19_ control antibody responses for these samples (> 27,000 MFI-bg units), it is possible that the competitor protein concentration was insufficient for complete antibody blocking. As previously demonstrated, addition of GST control protein to the competition assay at a concentration of 10 μg/ml had no effect on the antibody responses.

One sample (Mozambique 1, Table 4) demonstrated a partial loss (40% reduction) of anti-*P. malariae* MSP1_19_ antibody response in the presence of *P. falciparum* competitor protein, but antibodies to the other 3 species antigens were specific. Four samples, represented by Mozambique 16 in Table 4, demonstrated some combination of incomplete homologous response suppression and heterologous assay inhibition. In the case of Mozambique 16, the *P. falciparum* competitor protein partially inhibited the heterologous *P. malariae* MSP1_19_ antibody response (68% reduction), and the *P. vivax* competitor protein had a major impact on the *P. ovale* heterologous response (99% reduction), but, in the same reaction, this competitor did not completely block the homologous *P. vivax* MSP1_19_ antibody response (90% reduction). Reciprocal heterologous competition was only observed between *P. ovale* and *P. vivax* MSP1_19_ antigens and only in two donors represented by Mozambique 19 (Table 4). Heterologous competition assays leading to response values below the cut-off were observed for both *P. malariae* and *P. vivax* MSP1_19_ proteins and the *P. ovale* antigen partially reduced the *P. vivax* antibody response.

As shown in Table 5, what appeared to be a true, pan-malaria MSP1_19_ antibody response was observed in blood spot elutions from two 20–30 years old Mozambique donors, numbers 3 and 12. In contrast to assays described above, control response values to MSP1_19_ proteins for 3 of the malaria species (*P. malariae*, *P. ovale*, *P. vivax*) were reduced by about 50% upon addition of GST control protein. The reason for the observed signal suppression by GST is not understood, but it was probably not related to the presence of the GST component of the MSP1_19_-GST fusion proteins since no antibody bound to the GST control bead and there was no decrease in the PfCSP peptide-GST response. For both donors, the response to the *P. falciparum* MSP1_19_ antibody response was less affected by the addition of the GST control protein, and residual *P. falciparum* MSP1_19_ antibody signal (6–9% of control) was observed in the presence of each of the heterologous competitor proteins. Antibody responses to the other 3 MSP1_19_ proteins in the presence of heterologous competitor proteins were either below or very near the cut-off values for the respective assays (Table 5).

**Table 5:** Mozambique blood spot elutions that demonstrate multiple cross-reacting MSP1_19_ antibody responses

Sample	Competitor added	Pf MSP1_19_ (MFI-bg)	Pm MSP1_19_ (MFI-bg)	Po MSP1_19_ (MFI-bg)	PvMSP1_19_ (MFI-bg)	PfCSP (MFI-bg)	GST (MFI-bg)
Mozambique 3	PBS buffer only	24,960	13,703	11,965	21,795	23,643	4
	GST	19,926	***6961***	***6418***	***12,687***	23,442	2
	Pf MSP1_19_	*40* ^a^	237 ^c^	53	***232***	23,742	1
	Pm MSP1_19_	***2211*** ^b^	*17*	40	***210***	23,283	2
	Po MSP1_19_	***2487***	183	*27*	***227***	23,919	3
	Pv MSP1_19_	***2506***	232	69	*40*	23,882	1
Mozambique 12	PBS buffer only	24,121	13,890	9743	10,178	25,839	20
	GST	22,343	***6172***	***4284***	***4944***	25,547	10
	Pf MSP1_19_	*62*	62	36	175	25,874	10
	Pm MSP1_19_	***1420***	*22*	31	156	25,062	6
	Po MSP1_19_	***1488***	54	*31*	172	25,492	7
	Pv MSP1_19_	***1726***	75	49	*30*	25,447	8

^a^Multiplex bead assay responses that were completely suppressed by homologous protein competition are indicated in italics cells. These responses were below the respective cut-off values

^b^Multiplex bead assay responses that, as a result of heterologous competition, decreased more than 30% relative to the PBS control but remained above the respective cut-off values are indicated in bold italics cells. These values indicate some level of cross-reactivity

^c^Multiplex bead assay responses that were completely suppressed by heterologous protein competition are indicated in underlined cells. These responses were below the respective cut-off values

### Affinity purification of MSP1_19_ antibodies

Another potential method to detect antibody cross-reactivity is to affinity purify antibody using an MSP1_19_ protein from a single malaria species and then assess the reactivity of the eluted antibody using MSP1_19_ coated beads from all 4 species. Tetanus toxoid, a protein lacking GST, was included in the multiplex panel as an additional assay control.

*Plasmodium vivax* MSP1_19_-GST-coated magnetic beads were used to affinity purify antibodies from two Mozambique samples: sample 13, previously shown to have specific responses to all 4 MSP1_19_ proteins; and sample 16, previously shown to have significant cross-reactivity between the *P. vivax* protein and the *P. malariae* antibody response (Table 4). In the case of Mozambique sample 13, only the antibody response to the *P. vivax* MSP1_19_ decreased in the serum dilution after exposure to the magnetic beads (Table 6), and antibodies eluted from the magnetic bead only reacted with the *P. vivax* bead in the multiplex assay. In a separate experiment with sample 13, captured *P. malariae* antibodies were eluted from beads coated with the homologous antigen (Additional file 4). For sample 16, antibody responses to both the *P. ovale* and the *P. vivax* MSP1_19_ proteins decreased upon exposure of the serum dilution to *P. vivax* protein-coated magnetic beads, and eluted antibodies reacted to both *P. vivax* and *P. ovale* beads in the multiplex assay. Thus, the cross-reactivity previously observed in the competition assays was confirmed for this sample.

**Table 6:** Affinity purification of MSP1_19_ binding antibodies using magnetic bead capture

Sample	Description	Pf MSP1_19_ (MFI-bg)	Pm MSP1_19_ (MFI-bg)	Po MSP1_19_ (MFI-bg)	Pv MSP1_19_ (MFI-bg)	PfCSP (MFI-bg)	GST (MFI-bg)	Tet^a^ (MFI-bg)
Mozambique 13	No treatment	29,650	26,475	1445	28,371	26,857	12	2424
	Post incubation with Pv coated beads	30,363	26,631	1612	15,734	28,008	13	3517
	Antibody eluted from Pv coated beads	4	0	2	*1061* ^b^	0	1	2
Mozambique 16	No treatment	28,323	6713	18,227	24,238	18,909	28	24,114
	Post incubation with Pv coated beads	29,956	7588	3001	15,417	22,871	34	26,239
	Antibody eluted from Pv coated beads	0	3	***229*** ^c^	*2173*	0	0	0
Mozambique 15	No treatment	28,351	1555	17,238	264	25,020	7	12,797
	Post incubation with Pf coated beads	12,216	1928	20,876	328	28,102	7	16,773
	Antibody eluted from Pf coated beads	*4267*	2	0	34	0	0	0
Mozambique 12	No treatment	25,821	15,187	10,551	11,465	27,920	38	25,409
	Post incubation with Pf coated beads	3919	1213	806	1538	29,536	37	27,357
	Antibody eluted from Pf coated beads	84	28	21	10	0	1	1

^a^Tetanus toxoid, a protein lacking GST, was used as an additional control

^b^Positive antibody responses recognizing the same MSP1_19_ antigen as that used in the capture assay are indicated in italics

^c^Positive antibody responses recognizing an MSP1_19_ antigen not used in the capture assay are indicated in bold italics

*Plasmodium falciparum* MSP1_19_-GST-coated magnetic beads were used to affinity purify antibodies from two additional Mozambique samples: sample 15, previously shown to have specific responses to all 4 MSP1_19_ proteins (Table 4); and sample 12, previously shown to have a pan-malaria cross-reactive response (Table 5). As expected for a species-specific antibody response, only the *P. falciparum* MSP1_19_ antibody response decreased in the serum dilution following exposure to magnetic beads, and the elution only contained antibodies that recognized the *P. falciparum* protein in the multiplex assay (Table 6). Incubation of Mozambique sample 12 with the *P. falciparum* MSP1_19_-GST-coated magnetic beads drastically decreased the antibody responses to proteins from all 4 species in the post-treatment serum dilution, but positive responses were not observed in the MBA analysis of the eluted antibodies. *Plasmodium ovale* MSP1_19_ –GST-coated magnetic beads were also used for antibody capture from sample 12 with results similar to those described above (Additional file 4).

### Sub-class and avidity studies

The inability to affinity purify and recover antibodies from a highly cross-reactive sample suggested that the antibody response in Mozambique 12 might have some unique features relative to responses that were species specific or weakly cross-reactive. Table 7 shows that, while the Mozambique sample 13 anti-MSP1_19_ antibody responses were predominantly of the IgG_1_ sub-class, Mozambique sample 16 and 15 responses were a combination mainly of IgG_1_, IgG_2_, and IgG_3_ sub-classes. Of particular interest, the *P. falciparum* and *P. malariae* MSP1_19_ responses for both donors had strong IgG_3_ components, but the *P. ovale* and *P. vivax* responses for sample 16 were largely of the IgG_2_ sub-class. Further, the MSP1_19_ antibody responses observed in Mozambique 13, 16 and 15 samples appeared to be a mixture of high avidity and low avidity antibodies as determined by the 6 M urea treatment, with responses to the *P. falciparum* MSP1_19_ having a high avidity (≥ 0.98) and responses to the *P. vivax* MSP1_19_ being mainly low avidity (ratio ≤ 0.12). This low avidity, however, did not prevent antibody capture and elution using *P. vivax* MSP1_19_-coated beads shown in Table 6.

**Table 7:** IgG sub-class and antibody avidity index for samples from Mozambique

Sample	Antibody detected	Description	Pf MSP1_19_ (MFI-bg)	Pm MSP1_19_ (MFI-bg)	Po MSP1_19_ (MFI-bg)	Pv MSP1_19_ (MFI-bg)	PfCSP (MFI-bg)	GST (MFI-bg)	Tet (MFI-bg)
Mozambique 13	IgG_1_	No treatment	25,380	7236	369	23,068	7081	1	700
	IgG_2_	No treatment	1255	214	36	778	21,283	3	17
	IgG_3_	No treatment	404	441	50	281	7555	0	10
	IgG_4_	No treatment	26	4	3	9	0	0	28
	Total IgG	No treatment	31,255	26,542	1198	30,077	28,809	12	1861
	Total IgG	6 M urea wash	31,009	5724	623	2297	28,005	3	2253
	Total IgG	Avidity index^a^	*0.99*	*0.22*	*0.52*	*0.08*	*0.97*	*N/D*	*1.21*
Mozambique 16	IgG_1_	No treatment	2425	627	89	190	3722	9	5100
	IgG_2_	No treatment	107	41	6003	22,534	205	4	168
	IgG_3_	No treatment	30,533	3401	43	55	5654	2	107
	IgG_4_	No treatment	52	6	7	7	13	0	1206
	Total IgG	No treatment	30,369	59,12	16,287	23,777	20,145	35	24,367
	Total IgG	6 M Urea wash	29,850	3456	1689	223	7332	4	24,683
	Total IgG	Avidity index	*0.98*	*0.58*	*0.10*	*0.01*	*0.36*	*N/D*	*1.01*
Mozambique 15	IgG_1_	No treatment	14,698	416	4196	89	11,752	1	2348
	IgG_2_	No treatment	573	18	58	26	578	4	67
	IgG_3_	No treatment	30,026	2400	48	64	19,941	0	86
	IgG_4_	No treatment	3271	9	4	3	281	0	2934
	Total IgG	No treatment	30,749	1652	18,046	302	28,269	8	14,066
	Total IgG	6 M Urea wash	30,298	1183	6032	35	25,232	2	13,336
	Total IgG	Avidity index	*0.99*	*0.72*	*0.33*	*0.12*	*0.89*	*N/D*	*0.95*
Mozambique 12	IgG_1_	No treatment	269	45	35	57	7043	2	4212
	IgG_2_	No treatment	28	15	26	15	468	5	188
	IgG_3_	No treatment	24,515	11,988	8263	8259	22,011	3	33
	IgG_4_	No treatment	6	1	2	3	9	0	177
	Total IgG	No treatment	23,607	6741	4437	4162	26,444	16	21,638
	Total IgG	6 M Urea wash	719	36	29	61	19,765	4	19,375
	Total IgG	Avidity index	*0.03*	*0.01*	*0.01*	*0.01*	*0.75*	*N/D*	*0.90*

^a^The total IgG antibody avidity index, indicated in italic cells, was calculated by dividing the total IgG response after 6 M urea wash by the total IgG response with no treatment

*N/D* not determined

The pan-malaria cross-reactive response of Mozambique 12 was completely different. The response to MSP1_19_ proteins from all four malaria species was almost exclusively of the IgG_3_ sub-class, and the entire IgG response was low avidity with avidity index values of 0.01–0.03 (Table 7). The donor was clearly capable of making high avidity IgG antibodies of other sub-classes as evidenced by the responses to the *P. falciparum* CSP and tetanus toxoid (Table 7). Unfortunately, this observation could not be confirmed using the other highly cross-reactive sample (Mozambique 3) as no additional antibody eluate was available for testing.

## Discussion

Serologic antibody responses to malaria MSP1_19_ antigens are increasingly used to map geographic distributions and transmission intensities of malaria infection, but questions about the specificity of the responses remain incompletely explored [17–19]. Genomic sequence analysis demonstrates limited allelic variability within species (often only 2–3 amino acids), but considerable sequence heterogeneity between species (this work [35, 38, 65, 66, 67]). In a recent serologic IgG antibody survey of two communities in northern Mozambique [41], a non-trivial 2–4% prevalence for IgG antibodies to *P. vivax* MSP1_19_ antigen was observed in a population that is expected to be ≥ 95% negative for the Duffy marker used for RBC invasion [10–12, 70]. The current study was undertaken to determine whether these unexpected responses represented antibody cross-reactivity resulting from the transmission of *P. malariae* and *P. ovale* in the context of intense *P. falciparum* infection or whether they represented true *P. vivax* infections [41].

First, monoplex bead assays using a single MSP1_19_ antigen were compared to multiplex bead assays that included beads coated with antigens from all 4 species as well as GST control and PfCSP peptide coupled to GST. The monoplex *versus* multiplex results for all 4 MSP1_19_ antigens using a panel of 2 sera and 4 blood spot elutions with a range of antibody response values were virtually identical, and no response dilution effect was detected. Similar results were previously reported by Kerkhof et al. [31] using the *P. falciparum* and *P. vivax* MSP1_19_ antigens and 3 different positive control serum dilutions. However, the observation that a two-fold increase in the number of beads used per assay well had only marginal effects on the measured *P. falciparum* and *P. vivax* MSP1_19_ antibody responses [31] suggests that this technique may not be a sensitive method for identifying partial cross-reactivity.

Second, banked sera from chimpanzees infected with a single species of malaria in a controlled laboratory setting were tested by MBA. While all 4 animals had homologous antibody responses to the laboratory-administered parasite infection, 2 of the animals also had weak heterologous antibody responses to the *P. vivax* antigen and strong heterologous responses to the *P. falciparum* antigen despite the fact that they had never been infected with either of these parasites in the laboratory. Responses to the PfCSP antigen were also observed despite the lack of laboratory exposure to *P. falciparum* sporozoites. The presence of the *P. falciparum* MSP1_19_ and CSP responses strongly suggested that infections had occurred in the wild, perhaps with one of the Laveranian great ape malaria species that are genetically very similar to *P. falciparum* [69]. Muerhoff et al. came to the same conclusion regarding a *P. falciparum* MSP1_19_ response observed in chimpanzee sera by ELISA [36]. The absence of pre-exposure baseline sera for the chimpanzees meant that it was impossible to discriminate between cross-reactive responses resulting from the laboratory infections and pre-existing responses from infections acquired in the wild prior to capture.

Suppression of antibody binding by pre-absorption with excess heterologous or homologous MSP1_19_ antigen should be a sensitive method for the identification of cross-reactive antibody responses in MBA. Amanfo et al. [42] used 2 sera with a competition ELISA technique to demonstrate a lack of cross-reactivity between *P. falciparum*, *P. ovale* and *P. malariae* MSP1_19_ antigens (the *P. vivax* antigen was not included in their analysis). In the third part of this current study, 12 samples from low transmission areas in Haiti and Cambodia and 20 samples from a high transmission area in Mozambique were used to assess cross-reactivity by antigen competition MBA. Eleven of 12 sera from residents of the low transmission areas had MSP1_19_ antibody responses that were completely species specific. Only one individual had a heterologous competition response decrease that met the > 30% reduction definition. In the Mozambique sample set, antibody responses for 12 of the 20 samples tested were totally species specific, and 6 of these 12 samples were positive for antibodies to all 4 malaria parasite species. For 6 additional Mozambique samples, the *P. falciparum* MSP1_19_ responses were species specific, but various levels of incomplete heterologous competition were observed for the non-*P. falciparum* assays ranging from a 31 to 99% response reduction. The high specificity of the *P. falciparum* assay may reflect the affinity maturation of the immune response upon repeated infection with *P. falciparum* in the high intensity transmission setting of Mozambique. Most heterologous competition was non-reciprocal, suggesting that infection with one malaria species elicited both specific and cross-reactive antibodies while infection with the other malaria species resulted in only specific antibodies. Most commonly, *P. malariae* responses cross-reacted with *P. falciparum* antigen. Two examples of reciprocal heterologous competition were also identified, and both of these involved *P. vivax* and *P. ovale* responses. Whether higher concentrations of competitor MSP1_19_-GST protein (> 10 μg/ml) might have resulted in more complete heterologous competition of these responses was not determined.

Two individuals were identified who had very high responses to all 4 MSP1_19_ antigens (> 9000 MFI-bg units) and who appeared to have pan-malaria MSP1_19_ antibody responses by antigen competition MBA. However, perhaps because of the very high levels of antibodies generated by intense levels of *P. falciparum* transmission, heterologous antigens could only partially suppress the *P. falciparum* antibody response. These 2 samples represent only 15% of the 13 samples that were positive for antibodies to all four malaria species in the high transmission area sample set, and it should be noted that the sample set was not randomly selected from the overall Mozambique bed net study population. In fact, samples with high responses to the non-*P. falciparum* MSP1_19_ antigens were intentionally chosen in an attempt to identify those ‘worst case scenario’ samples where cross-reactivity might be observed. Because only 40 samples from the overall Mozambique bed net study set (*N *= 2408) had responses above the cut-off values to all 4 MSP1_19_ antibodies [41], the number of potential pan-malaria reactive individuals in Mozambique is likely quite low (< 0.3%).

Finally, an antibody capture/elution technique was used with the MBA to confirm the results of the competition assays described above. While species specific and partially cross-reactive MSP1_19_ antibodies could be eluted from capture beads, appreciable quantities of captured antibodies could not be recovered from the pan-malaria responsive DBS elution despite repeated attempts with multiple capture antigens. Further analysis of the samples from the species specific and partially cross-reactive donors revealed IgG responses of varying avidity dominated by the IgG_1_ and IgG_3_ sub-classes. Others have reported that exposure to *P. falciparum* MSP1_19_ elicits a mixed pattern of IgG_1_ and IgG_3_ antibodies and that repeated infection causes a shift towards an IgG_1_-dominated response [35, 71–78]. The species specific and partially cross-reactive results presented here are consistent with those reports. In contrast, the pan-malaria response from Mozambique sample 12 exhibited very low avidity binding to MSP1_19_ antigens from all 4 malaria species and was skewed entirely to the IgG_3_ sub-class. Low avidity responses to the *P. falciparum* MSP1_19_ are relatively rare [74], and only one previously reported example of a mixed IgG_1_/IgG_3_ response that skewed almost entirely to an IgG_3_ response upon repeat infection with *P. falciparum* was found in the literature [78]. At present, it cannot be determined whether these observations result from host-specific factors or are a universal feature of pan-malaria responses, nor can any definitive conclusions be drawn about the impact of such responses on malaria immunity or potential malaria pathology.

Previous studies on allele-specific antibody responses to *P. falciparum* MSP1_19_ and apical membrane antigen 1 (AMA1) suggested that children develop allele-specific responses upon primary infection and that the prevalence of cross-reactive antibodies to conserved epitopes increases with age and increasing experience of infection [79, 80]. The number of samples in this study was too small to definitively address the issue of age as a proxy for infection experience and the development of cross-reactive antibody responses. However, two of the partially cross-reactive samples from Mozambique were from 5-years old donors, and 5 of the donors with specific antibody responses to all 4 malaria species were > 50 years of age. Simultaneous infection with multiple malaria species, which is known to occur in Mozambique [81], might play a larger role in the development of antibody responses against shared MPS1_19_ epitopes than total infection experience [82]. Thus, even in a high transmission setting with multiple co-endemic malaria species, most antibody responses to *P. falciparum*, *P. malariae*, *P. ovale*, and *P. vivax* MSP1_19_ antigens are likely indicative of previous infection history with those parasite species.

## Conclusions

Globally, most areas of malaria transmission are seldom mono-specific. In sub-Saharan Africa, *P. falciparum* is the most prevalent infection with the highest intensity of transmission, but significant transmission attributable to *P. malariae*, *P. ovale,* and, in some areas, *P. vivax* occurs. In South and Central America, *P. malariae*, *P. falciparum,* and *P. vivax* are transmitted endemically whereas in Asia all four human malaria species can be transmitted. MSP-1_19_ is a major antigen recognized by the IgG antibody response of a majority of exposed individuals in an endemic population. Malaria control efforts would likely benefit from being able to rapidly and easily monitor immune responses not only for the main targeted species such as *P. falciparum* and *P. vivax,* but also the lesser species, *P. malariae* and *P. ovale*. The analyses presented in this work indicate that the multi-species MSP-1_19_ multiplex bead assay will be a useful tool in future malaria epidemiologic surveillance and control program studies.

### Footnotes

Use of trade names is for identification only and does not imply endorsement by the Public Health Service or by the US Department of Health and Human Services. The findings and conclusions in this report are those of the authors and do not necessarily represent the official position of the Centers for Disease Control and Prevention.

## Additional files

**Additional file 1.** Human antibody competition titration assays using MSP1_19_ proteins from four *Plasmodium* species. A combined dilution (1:400 of each serum) containing sera from pan *Plasmodium* Lot 8 and *P. malariae* Lot 2 defined human sera was incubated with the indicated concentrations of the MSP1_19_ competitor protein for 1 hr at room temperature. Competitor proteins used were: Panel A, *P. falciparum* MSP1_19_; Panel B, *P. malariae* MSP1_19_; Panel C, *P. ovale* MSP1_19_; Panel D, *P. vivax* MSP1_19_. Multiplex bead assays were performed as described in “Methods” and the multiplex response in MFI-bg units are plotted *versus* the competitor concentration. Multiplex responses are presented as a percentage of the assay results for the PBS control.

**Additional file 2.** Additional MSP1_19_ competition assay results using sera from low malaria incidence settings.

**Additional file 3.** Additional MSP1_19_ competition assay results using sera from a high malaria incidence setting.

**Additional file 4.** Additional MSP1_19_ antibody binding and elution assays using beads coated with *Plasmodium ovale* (Po) or *Plasmodium malariae* (Pm) antigens.

## Abbreviations

MBA: multiplex bead assay
MSP1_19_: 19 kDa subunit of merozoite surface protein 1
CSP: circumsporozoite protein
IFA: immunofluorescence assay
PVA: polyvinyl alcohol
PVP: polyvinylpyrrolidone
PBS: buffer containing 10 mM Na_2_HPO_4_ and 0.85% NaCl at pH 7.2
Buffer A: PBS buffer (pH 7.2) containing 0.3% Tween-20, 0.02% NaN_3_, 0.5% casein, 0.5% PVA, 0.8% PVP, and 3 μg/ml *E. coli* extract
Buffer B: PBS buffer (pH 7.2) containing 0.5% BSA, 0.05% Tween-20, and 0.02% NaN_3_
Tris: tris(hydroxymethyl)-aminomethane
GST: *S. japonicum* glutathione-*S*-transferase
MES: 2-(*N*-morpholino)-ethanesulfonic acid
HEPES: *N*-hydroxyethylpiperazine-*N*’-2-ethanesulfonic acid
MFI-bg: median fluorescent intensity *minus* background
MES: 2-(*N*-morpholino)-ethanesulfonic acid
Pm: *P. malariae*
Po: *P. ovale*
Pv: *P. vivax*
Pf: *P. falciparum*
AMA1: apical membrane protein 1
