# Supplementary figures and images for: Specificity of the IgG antibody response to Plasmodium falciparum, Plasmodium vivax, Plasmodium malariae, and Plasmodium ovale MSP119 subunit proteins in multiplexed serologic assays
Source: Malar J. 2018 Nov 9;17:417. doi: 10.1186/s12936-018-2566-0 (PMC6230236; doi:10.1186/s12936-018-2566-0)

## Slide 1
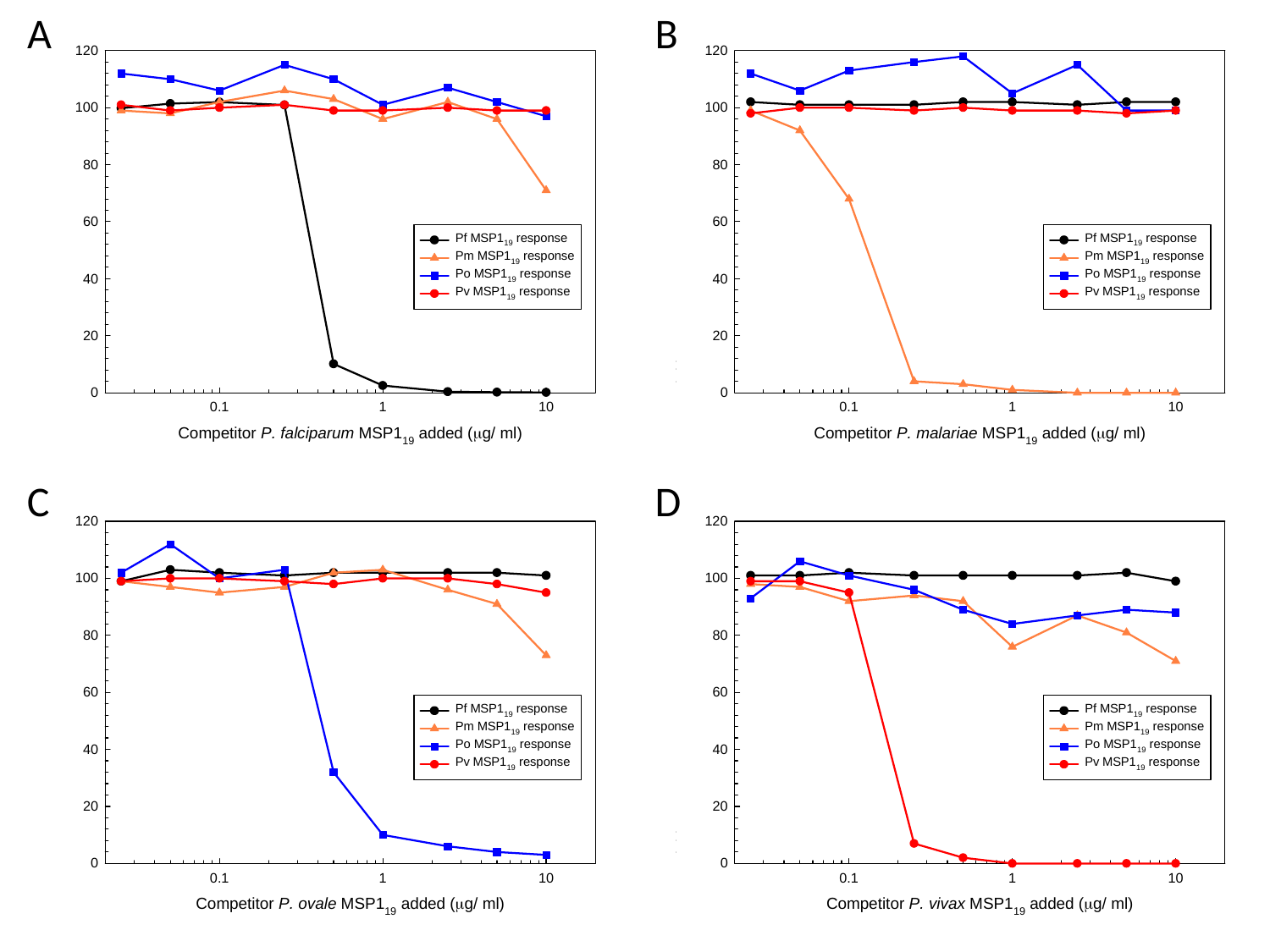

A
B
C
D

Supplement: Supplementary file 1 — Additional file 1. Human antibody competition titration assays using MSP119 proteins from four Plasmodium species. A combined dilution (1:400 of each serum) containing sera from pan Plasmodium Lot 8 and P. malariae Lot 2 defined human sera was incubated with the indicated concentrations of the MSP119 competitor protein for 1 hr at room temperature. Competitor proteins used were: Panel A, P. falciparum MSP119; Panel B, P. malariae MSP119; Panel C, P. ovale MSP119; Panel D, P. vivax MSP119. Multiplex bead assays were performed as described in “Methods” and the multiplex response in MFI-bg units are plotted versus the competitor concentration. Multiplex responses are presented as a percentage of the assay results for the PBS control. [file 12936_2018_2566_MOESM1_ESM.pptx]
