# Supplementary material for: Specificity of the IgG antibody response to Plasmodium falciparum, Plasmodium vivax, Plasmodium malariae, and Plasmodium ovale MSP119 subunit proteins in multiplexed serologic assays
Source: Malar J. 2018 Nov 9;17:417. doi: 10.1186/s12936-018-2566-0 (PMC6230236; doi:10.1186/s12936-018-2566-0)
Supplement: Supplementary file 2 — Additional file 2. Additional MSP119 competition assay results using sera from low malaria incidence settings. [file 12936_2018_2566_MOESM2_ESM.docx]

|  | **Competitor** | **Pf MSP1_19_** | **Pm MSP1_19_** | **Po MSP1_19_** | **PvMSP1_19_** | **PfCSP** | **GST** |
| --- | --- | --- | --- | --- | --- | --- | --- |
| **Sample** | **Added** | **(MFI - bg)** | **(MFI - bg)** | **(MFI - bg)** | **(MFI - bg)** | **(MFI - bg)** | **(MFI - bg)** |
| Haiti 2 | PBS buffer only | 26031 | 13 | 10 | 19 | 20 | 0 |
|  | GST | 25760 | 11 | 11 | 21 | 18 | 2 |
|  | Pf MSP1_19_ | 8 | 13 | 9 | 22 | 20 | 0 |
|  | Pm MSP1_19_ | 25344 | 4 | 6 | 9 | 18 | 1 |
|  | Po MSP1_19_ | 25870 | 7 | 4 | 9 | 18 | 1 |
|  | Pv MSP1_19_ | 25871 | 7 | 6 | 2 | 17 | 1 |
|  |  |  |  |  |  |  |  |
| Haiti 3 | PBS buffer only | 24556 | 9 | 23 | 9 | 56 | 2 |
|  | GST | 24435 | 10 | 20 | 7 | 54 | 3 |
|  | Pf MSP1_19_ | 11 | 9 | 23 | 7 | 51 | 2 |
|  | Pm MSP1_19_ | 24462 | 7 | 19 | 7 | 56 | 2 |
|  | Po MSP1_19_ | 24129 | 6 | 5 | 6 | 47 | 3 |
|  | Pv MSP1_19_ | 24161 | 9 | 19 | 4 | 54 | 2 |
|  |  |  |  |  |  |  |  |
| Cambodia 1 | PBS buffer only | 30146 | 15 | 77 | 126 | 881 | 6 |
|  | GST | 30221 | 15 | 92 | 142 | 780 | 1 |
|  | Pf MSP1_19_ | 25 | 12 | 71 | 95 | 770 | 7 |
|  | Pm MSP1_19_ | 30211 | 4 | 77 | 127 | 774 | 7 |
|  | Po MSP1_19_ | 30281 | 9 | 8 | 57 | 845 | 9 |
|  | Pv MSP1_19_ | 30369 | 11 | 73 | 3 | 888 | 9 |
|  |  |  |  |  |  |  |  |
| Cambodia 6 | PBS buffer only | 30 | 4 | 5 | 27772 | 14 | 0 |
|  | GST | 37 | 3 | 5 | 28281 | 16 | 0 |
|  | Pf MSP1_19_ | 23 | 4 | 7 | 28156 | 15 | 1 |
|  | Pm MSP1_19_ | 28 | 1 | 5 | 28066 | 14 | 0 |
|  | Po MSP1_19_ | 27 | 2 | 1 | 27903 | 14 | 0 |
|  | Pv MSP1_19_ | 30 | 3 | 6 | 14 | 15 | 0 |
|  |  |  |  |  |  |  |  |
| Cambodia 7 | PBS buffer only | 26704 | 536 | 10 | 22 | 3930 | 2 |
|  | GST | 26852 | 524 | 10 | 23 | 3727 | 2 |
|  | Pf MSP1_19_ | 23 | 509 | 6 | 15 | 3900 | 2 |
|  | Pm MSP1_19_ | 26630 | 5 | 10 | 10 | 3737 | 1 |
|  | Po MSP1_19_ | 26755 | 536 | 6 | 23 | 3662 | 1 |
|  | Pv MSP1_19_ | 26729 | 488 | 12 | 3 | 3657 | 3 |
|  |  |  |  |  |  |  |  |
| Cambodia 8 | PBS buffer only | 27161 | 1382 | 9 | 37 | 435 | 5 |
|  | GST | 27299 | 1231 | 10 | 40 | 432 | 4 |
|  | Pf MSP1_19_ | 12 | 1339 | 8 | 17 | 525 | 5 |
|  | Pm MSP1_19_ | 27334 | 6 | 8 | 31 | 511 | 5 |
|  | Po MSP1_19_ | 27315 | 1333 | 8 | 37 | 459 | 5 |
|  | Pv MSP1_19_ | 27208 | 1085 | 10 | 6 | 494 | 5 |

Additional file 2: Table S1
