# Supplementary material for: Specificity of the IgG antibody response to Plasmodium falciparum, Plasmodium vivax, Plasmodium malariae, and Plasmodium ovale MSP119 subunit proteins in multiplexed serologic assays
Source: Malar J. 2018 Nov 9;17:417. doi: 10.1186/s12936-018-2566-0 (PMC6230236; doi:10.1186/s12936-018-2566-0)
Supplement: Supplementary file 3 — Additional file 3. Additional MSP119 competition assay results using sera from a high malaria incidence setting. [file 12936_2018_2566_MOESM3_ESM.docx]

Additional file 3: Table S2

|  | **Competitor** | **Pf MSP1_19_** | **Pm MSP1_19_** | **Po MSP1_19_** | **PvMSP1_19_** | **PfCSP** | **GST** |
| --- | --- | --- | --- | --- | --- | --- | --- |
| **Sample** | **Added** | **(MFI - bg)** | **(MFI - bg)** | **(MFI - bg)** | **(MFI - bg)** | **(MFI - bg)** | **(MFI - bg)** |
| Mozambique 5 | PBS buffer only | 28979 | 12448 | 634 | 514 | 24676 | 4 |
|  | GST | 28782 | 12680 | 604 | 514 | 24667 | 2 |
|  | Pf MSP1_19_ | 73 | 13166 | 544 | 483 | 24648 | 4 |
|  | Pm MSP1_19_ | 28844 | 21 | 575 | 513 | 24708 | 1 |
|  | Po MSP1_19_ | 28890 | 12778 | 41 | 564 | 24597 | 3 |
|  | Pv MSP1_19_ | 28688 | 12338 | 554 | 83 | 24353 | 2 |
|  |  |  |  |  |  |  |  |
| Mozambique 7 | PBS buffer only | 12230 | 1141 | 15082 | 144 | 23044 | 2 |
|  | GST | 12112 | 1121 | 15201 | 146 | 23199 | 2 |
|  | Pf MSP1_19_ | 20 | 1096 | 14472 | 129 | 22952 | 1 |
|  | Pm MSP1_19_ | 10863 | 13 | 15242 | 117 | 23104 | 1 |
|  | Po MSP1_19_ | 11206 | 1216 | 72 | 123 | 23341 | 3 |
|  | Pv MSP1_19_ | 10445 | 1034 | 14582 | 21 | 23076 | 1 |
|  |  |  |  |  |  |  |  |
| Mozambique 8 | PBS buffer only | 28592 | 24963 | 22 | 93 | 18714 | 3 |
|  | GST | 28666 | 25039 | 22 | 91 | 18944 | 1 |
|  | Pf MSP1_19_ | 60 | 25013 | 19 | 48 | 18580 | 1 |
|  | Pm MSP1_19_ | 28645 | 64 | 21 | 86 | 17086 | 1 |
|  | Po MSP1_19_ | 28723 | 25041 | 16 | 87 | 18637 | 2 |
|  | Pv MSP1_19_ | 28251 | 24906 | 17 | 17 | 18368 | 0 |
|  |  |  |  |  |  |  |  |
| Mozambique 9 | PBS buffer only | 29396 | 34 | 30 | 563 | 2897 | 3 |
|  | GST | 29662 | 31 | 26 | 689 | 2885 | 5 |
|  | Pf MSP1_19_ | 80 | 29 | 21 | 680 | 2991 | 5 |
|  | Pm MSP1_19_ | 29610 | 16 | 28 | 590 | 2925 | 5 |
|  | Po MSP1_19_ | 29831 | 31 | 22 | 655 | 3060 | 5 |
|  | Pv MSP1_19_ | 29545 | 28 | 27 | 14 | 3261 | 6 |
|  |  |  |  |  |  |  |  |
| Mozambique 14 | PBS buffer only | 29529 | 8012 | 363 | 27827 | 16621 | 8 |
|  | GST | 29491 | 7652 | 318 | 27854 | 15945 | 10 |
|  | Pf MSP1_19_ | 71 | 5827 | 256 | 28278 | 16356 | 9 |
|  | Pm MSP1_19_ | 29369 | 277 | 263 | 27060 | 15498 | 9 |
|  | Po MSP1_19_ | 29233 | 7485 | 48 | 27546 | 16183 | 9 |
|  | Pv MSP1_19_ | 29591 | 7681 | 288 | 55 | 16058 | 11 |
|  |  |  |  |  |  |  |  |
| Mozambique 15 | PBS buffer only | 28036 | 1551 | 17798 | 277 | 25468 | 7 |
|  | GST | 28203 | 1462 | 17352 | 238 | 25715 | 7 |
|  | Pf MSP1_19_ | 33 | 1508 | 17018 | 247 | 25728 | 6 |
|  | Pm MSP1_19_ | 28294 | 15 | 16711 | 246 | 25954 | 5 |
|  | Po MSP1_19_ | 27918 | 1508 | 16 | 269 | 25959 | 7 |
|  | Pv MSP1_19_ | 28265 | 1479 | 18047 | 39 | 25583 | 7 |
|  |  |  |  |  |  |  |  |
| Mozambique 10 | PBS buffer only | 30017 | 26427 | 1733 | 394 | 27033 | 4 |
|  | GST | 29746 | 26164 | 1696 | 404 | 26598 | 5 |
|  | Pf MSP1_19_ | 286 | 25977 | 1579 | 353 | 26931 | 6 |
|  | Pm MSP1_19_ | 29896 | 30 | 1282 | 336 | 26601 | 4 |
|  | Po MSP1_19_ | 29805 | 26379 | 36 | 376 | 26482 | 3 |
|  | Pv MSP1_19_ | 29730 | 25961 | 1643 | 11 | 26451 | 4 |
|  |  |  |  |  |  |  |  |
| Mozambique 2 | PBS buffer only | 5438 | 27232 | 139 | 406 | 2833 | 15 |
|  | GST | 5594 | 27315 | 143 | 429 | 3118 | 17 |
|  | Pf MSP1_19_ | 31 | 27459 | 130 | 476 | 2867 | 15 |
|  | Pm MSP1_19_ | 5515 | 1673 | 122 | 426 | 3089 | 16 |
|  | Po MSP1_19_ | 5635 | 27376 | 25 | 425 | 2715 | 18 |
|  | Pv MSP1_19_ | 5173 | 27259 | 134 | 30 | 2853 | 15 |
|  |  |  |  |  |  |  |  |
| Mozambique 4 | PBS buffer only | 28394 | 5503 | 16702 | 24105 | 23103 | 16 |
|  | GST | 28472 | 5402 | 16769 | 24260 | 23094 | 18 |
|  | Pf MSP1_19_ | 22 | 1421 | 16464 | 24362 | 23268 | 15 |
|  | Pm MSP1_19_ | 27892 | 43 | 16668 | 24390 | 23382 | 12 |
|  | Po MSP1_19_ | 28516 | 5199 | 120 | 11117 | 23079 | 15 |
|  | Pv MSP1_19_ | 28142 | 5286 | 209 | 696 | 23113 | 16 |
|  |  |  |  |  |  |  |  |
| Mozambique 11 | PBS buffer only | 28263 | 255 | 25773 | 120 | 16614 | 5 |
|  | GST | 28181 | 239 | 25846 | 118 | 15432 | 2 |
|  | Pf MSP1_19_ | 1232 | 124 | 7282 | 50 | 16087 | 3 |
|  | Pm MSP1_19_ | 28547 | 29 | 25206 | 65 | 16260 | 2 |
|  | Po MSP1_19_ | 28185 | 158 | 125 | 44 | 16382 | 4 |
|  | Pv MSP1_19_ | 28053 | 188 | 25544 | 50 | 15719 | 4 |
|  |  |  |  |  |  |  |  |
| Mozambique 18 | PBS buffer only | 28062 | 27938 | 304 | 1256 | 23451 | 2 |
|  | GST | 27930 | 28162 | 319 | 1215 | 23432 | 2 |
|  | Pf MSP1_19_ | 33 | 27962 | 282 | 862 | 23568 | 1 |
|  | Pm MSP1_19_ | 27871 | 4770 | 240 | 695 | 23497 | 1 |
|  | Po MSP1_19_ | 27757 | 28057 | 21 | 1015 | 23600 | 1 |
|  | Pv MSP1_19_ | 28066 | 27753 | 265 | 58 | 23444 | 2 |
