# Supplementary material for: Specificity of the IgG antibody response to Plasmodium falciparum, Plasmodium vivax, Plasmodium malariae, and Plasmodium ovale MSP119 subunit proteins in multiplexed serologic assays
Source: Malar J. 2018 Nov 9;17:417. doi: 10.1186/s12936-018-2566-0 (PMC6230236; doi:10.1186/s12936-018-2566-0)
Supplement: Supplementary file 4 — Additional file 4. Additional MSP119 antibody binding and elution assays using beads coated with Plasmodium ovale (Po) or Plasmodium malariae (Pm) antigens. [file 12936_2018_2566_MOESM4_ESM.docx]

Additional file 4: Table S3

Affinity purification of MSP1_19_ binding antibodies using magnetic bead capture.

|  |  | **Pf MSP1_19_** | **Pm MSP1_19_** | **Po MSP1_19_** | **Pv MSP1_19_** | **PfCSP** | **GST** | **Tet^a^** |
| --- | --- | --- | --- | --- | --- | --- | --- | --- |
| **Sample** | **Description** | **(MFI - bg)** | **(MFI - bg)** | **(MFI - bg)** | **(MFI - bg)** | **(MFI - bg)** | **(MFI - bg)** | **(MFI - bg)** |
| Mozambique 13 | No treatment^b^ | 29650 | 26475 | 1445 | 28371 | 26857 | 12 | 2424 |
|  | Post incubation with Pm coated beads | 30699 | 16802 | 1287 | 29921 | 29214 | 12 | 2642 |
|  | Antibody eluted from Pm coated beads | 54 | 1684^c^ | 20 | 11 | 0 | 1 | 0 |
|  |  |  |  |  |  |  |  |  |
| Mozambique 12 | No treatment^b^ | 25821 | 15187 | 10551 | 11465 | 27920 | 38 | 25409 |
|  | Post incubation with Po coated beads | 18980 | 2895 | 1912 | 2969 | 29758 | 32 | 27552 |
|  | Antibody eluted from Po coated beads | 292 | 99 | 77 | 48 | 2 | 1 | 0 |

^a^Tetanus toxoid, a protein lacking GST, was used as an additional control.

^b^Values from Table 1.

^c^Positive antibody responses recognizing the same MSP1_19_ antigen as that used in the capture assay are indicated in green.
